# Development and preliminary evaluation of a suicidal risk assessment protocol in a randomised controlled trial using the Patient Health Questionnaire (PHQ-9)

**DOI:** 10.1186/s13063-024-08276-6

**Published:** 2024-07-12

**Authors:** Vari Wileman, Serena McGuinness, Louise Sweeney, Christine Norton, Laura Miller, Imogen Stagg, Ronan O’Carroll, Rona Moss-Morris

**Affiliations:** 1 Institute of Psychiatry, Psychology and Neuroscience, King’s College London, London, UK; 2 Florence Nightingale Faculty of Nursing, Midwifery & Palliative Care, King’s College London, London, UK; 3 Barts and the London Pragmatic Clinical Trials Unit, Queen Mary University of London, London, UK; 4 St Mark’s Hospital, The National Bowel Hospital and Academic Institute, London North West Hospitals NHS Trust, London, UK; 5 Psychology, Faculty of Natural Sciences, University of Stirling, Stirling, UK

**Keywords:** PHQ-9, Self-harm, Suicide, Ideation, Depression, Trials, Risk-assessment, Long-term conditions, Protocol

## Abstract

**Background:**

Participants in research trials often disclose severe depression symptoms, including thoughts of self-harm and suicidal ideation, in validated self-administered questionnaires such as the Patient Health Questionnaire (PHQ-9). However, there is no standard protocol for responding to such disclosure, and the opportunity to support people at risk is potentially missed. We developed and evaluated a risk assessment protocol for the IBD-BOOST randomised controlled trial (ISRCTN71618461 09/09/2019).

**Methods:**

Participants completed the PHQ-9 at baseline and 6-month and 12-month follow-ups. The trial database automatically alerted the research team to risk assess participants. Trial researchers, trained in the protocol, contacted participants by telephone, completed the risk assessment, and signposted participants to appropriate professional services.

**Results:**

Seven hundred eighty participants were randomised in the trial; 41 required risk assessment. One participant declined assessment, so 40 risk assessments were completed. Twenty-four participants were assessed as low-risk and 16 participants as medium-risk, with 12 declaring previous suicide attempts. None were rated as high-risk. Trial participants expressed appreciation for being contacted, and all except two wished to receive information about professional support services. Trial risk assessors reported positive experiences of conducting the risk assessment with suggestions for improvement, which resulted in minor modifications to the protocol.

**Discussion:**

Our evaluation demonstrated that it was viable for a research trial team to successfully conduct a risk-assessment protocol for trial participants reporting thoughts of self-harm, with training and support from senior colleagues. Resources are required for training and delivery, but it is not unduly onerous. Trial participants appeared to find completing the assessment acceptable.

**Supplementary Information:**

The online version contains supplementary material available at 10.1186/s13063-024-08276-6.

## Background

Depression is a common and serious mental health problem with over 280 million people affected worldwide [1]. People with long-term physical health conditions (LTCs) are two to three times more likely than those without LTCs to experience depression, contributing to poorer quality of life and health outcomes [2]. Cohort studies inform that the incidence of depression, deliberate self-harm, and suicide deaths is increased in people diagnosed with inflammatory bowel disease (IBD) [3–5]. In the UK National Health Service (NHS), National Institute of Health and Care Excellence (NICE) guidelines recommend pathways to counselling services for people experiencing depression (e.g. NHS Talking Therapies [6] or mental health charities (e.g. Mind) and urgent specialist mental health services and hospital emergency care as well as pharmaceutical treatments when required [7, 8]).

Interventions aimed at supporting people living with LTCs, including IBD, are widely investigated in randomised controlled trials. People participating in such trials routinely complete self-administered questionnaires about their mood, potentially disclosing severe depression symptoms including thoughts of self-harm and suicidal ideation [9]. However, there is no standard protocol for responding to such disclosure in research trials [10], and the opportunity to signpost people at risk to support services is potentially missed. It is increasingly recognised that recording such disclosure in trial participant questionnaires without a protocol for responding and supporting participants is potentially unethical [11].

Depression screening questionnaires are commonly used throughout UK NHS healthcare settings [12], including the Patient Health Questionnaire-9 (PHQ-9), which is recommended in UK NICE guidelines [13]. This self-report measure consists of nine items which are based on the diagnostic criteria for depressive disorder in the Diagnostic and Statistical Manual of Mental Disorders (DSM-IV) [14]. Patients are asked to rate the extent to which they have been bothered by problems over the last 2 weeks. Items are scored 0–3 (0 = *Not at all*, 1 = *Several days*, 2 = *More than half the days*, 3 = *Nearly every day*). Total scores range from 0 to 27, with a cut off score of ≥ 10 indicating major depressive disorder [9, 15]. Item 9 in the scale specifically asks patients to disclose thoughts of self-harm or suicidal ideation ‘*Thoughts you would be better off dead or hurting yourself in some way*’ [16] and is a reliable predictor of future self-harm [17],validated in people with IBD [18].^1^

In addition to being widely used as a screening tool in primary care, the PHQ-9 is commonly used to measure depressive symptoms in intervention trials. The tool contains an item about self-harm and suicidal ideation which necessitates a response mechanism. Typically, research participants complete the PHQ-9, amongst a host of other measures, either on paper or online, and usually complete the questionnaire independently and remotely from the research trial team, minimising the opportunity for researchers to review responses and check if the respondent is safe. Where trial databases are employed, potentially these data may not be examined until months or even years later when the trial data analysis commences, prompting the need for research trials to implement safety protocols closer to questionnaire completion for trial participants potentially at risk. Although research ethics committees review and approve all research activities, including any risk from participant completed measures, there are currently no formal requirements to follow-up with participants’ responses or to have any protocols in place if suicidal or self-harm ideation is reported. It has previously been suggested that research ethics committees may require formal response protocols to be in place before trial commencement [10], but we are not aware of any universal requirements currently. However, in our experience, some individual ethics committees are now asking about such safeguarding issues and how and when trial teams will pick up expression of suicidal ideation and how, and how quickly, they will respond.

Concerns have also been raised, including by research ethics committees, as to whether asking participants about suicide may lead to an increased risk of suicidal thoughts and behaviours [20, 21]. However, a recent meta-analysis looking at ‘suicide-content exposure in research trials’ found a small reduction in suicide ideation post-exposure [22]. Furthermore, in addition to potentially minimising risk, there is an important ethical issue to be considered where, as research investigators developing interventions to help people living with physical and mental health symptoms, we have a duty of care to support trial participants by signposting them to appropriate services. It is also important to consider the resources available within research trials to manage assessment processes and the qualifications and experience of trial researchers who could potentially respond appropriately to self-harm and suicide ideation disclosure.

We developed and evaluated a risk assessment protocol [23, 24], for trial research psychologists to respond to participants who alerted an ‘at risk’ status, during a National Institute for Health Research (NIHR) funded randomised controlled trial (IBD-BOOST, ISRCTN71618461) [25]. The IBD-BOOST trial evaluated a digital self-management programme based on a cognitive behavioural framework for fatigue, pain, and faecal incontinence in IBD, to improve quality of life. Like other LTCs with high prevalence of co-morbid anxiety and depression, about 25% of people with IBD experience depression, which increases to about 30% when disease activity increases [26]. As the IBD-BOOST intervention was in part targeting emotional responses to living with IBD and its symptoms, it was important to include a measure of depressive symptoms at baseline and follow-up during the trial. Trial participants were randomly allocated to either receive the IBD BOOST intervention or care as usual (control group). Participants completed trial questionnaires at three time points during the trial (pre-randomisation and 6-month and 12-month follow-ups). This paper reports the evaluation of the PHQ*-*9 trial risk assessment protocol delivered throughout the trial with recommendations for future trial practice.

## Methods

### Aims

The aim of the PHQ-9 risk assessment protocol was threefold.

1. To identify at baseline those who may be at risk before commencing the trial, to check that undertaking a self-directed cognitive behavioural intervention programme, which includes asking participants to explore emotional responses and potentially distressing experiences, would not be harmful. Anyone identified as high-risk would not be entered into the trial
2. Identifying and recording PHQ-9 risk assessments at 6-month and 12-month follow-ups would inform the trial investigators of any unexpected potentially adverse events of the intervention
3. To respond appropriately to trial participants who disclosed thoughts of self-harm or suicide on the PHQ-9 item 9 at all time points by giving them information and signposting them to appropriate professional services, including people who were not included in the trial because deemed to be high risk

### Participants and study setting

Participants aged 18 years or over living in England, Scotland or Wales, were eligible with a diagnosis of IBD (self-reported as having been medically diagnosed with IBD including patients with an ileo-anal pouch or stoma no urgent symptoms, such as rectal bleeding, that necessitated medical care and would potentially impact their participation in the trial). Full trial inclusion/exclusion criteria are reported in the randomised controlled trial protocol [25]. Access to the online intervention via a computer or mobile device was necessary for the study which was conducted at National Health Service (NHS) hospital sites in England with IBD services and at King’s College London (KCL) and recruitment. Trial recruitment commenced January 2020 until July 2022.

### Risk assessment protocol development

**Fig. 1:**
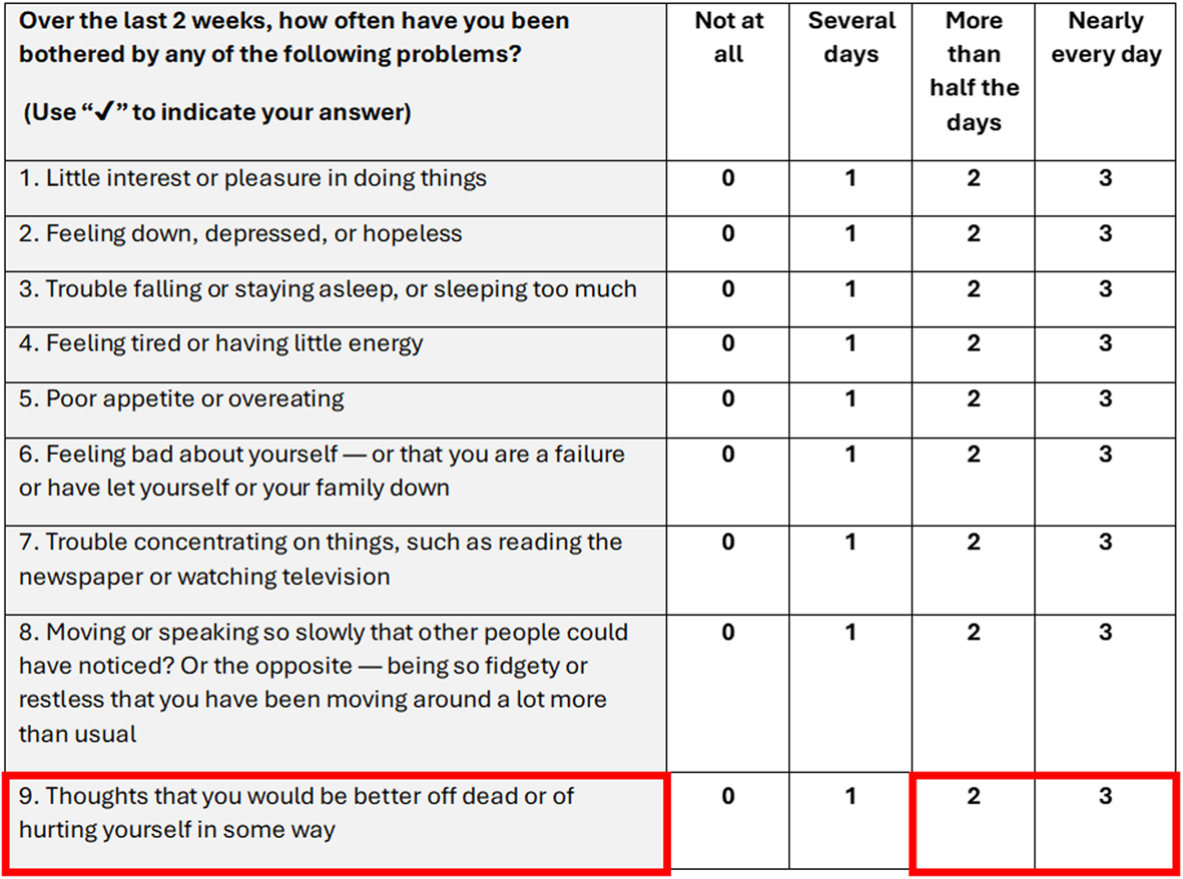
PHQ-9 scale items (▢ indicates item 9 responses which alerted risk assessment)

The trial research psychologists were not clinically responsible for participants, and we required a protocol that enabled researchers to check for participants’ safety and triage them to professional support services. Our protocol drew from Improving Access to Psychological Therapies (IAPT) UK service (now known as NHS Talking Therapies [6]) risk guidance and a risk assessment protocol used in an RCT of a digital intervention to treat distress (anxiety and depression) related to living with a long-term condition [23]. As people were participating in a research trial about their physical IBD symptoms and not necessarily seeking support for mental health distress, it was important to have an appropriate introductory script when calling participants unexpectedly about their self-harm responses, and we adapted the language for this purpose. We also altered the criteria at which we would risk assess participants to those who scored 2 ‘*More than half the days*’ or 3 ‘*Nearly every day*’ but not if they scored 1 ‘*Several days*’ (see Fig. 1). This decision was taken because the IBD-BOOST pain feasibility study found a high proportion of recruited participants expressed distress related to their IBD symptoms by scoring 1 ‘*Several days*’, rather than scoring 0 ‘*Not at all*’. The study found that participants who scored 1 explained that they were not actively expressing thoughts of self-harm or suicidal ideation, and researchers advised that risk assessment be applied to the higher scores of 2 or 3 [24]. The frequency of self-harm thoughts has also been associated with increased risk of suicide and a score of 2 or 3 has also been used in eligibility criteria for a population-based intervention study (*n* = 19,500) to prevent suicide attempt [27].

### Risk assessment protocol

**Fig. 2:**
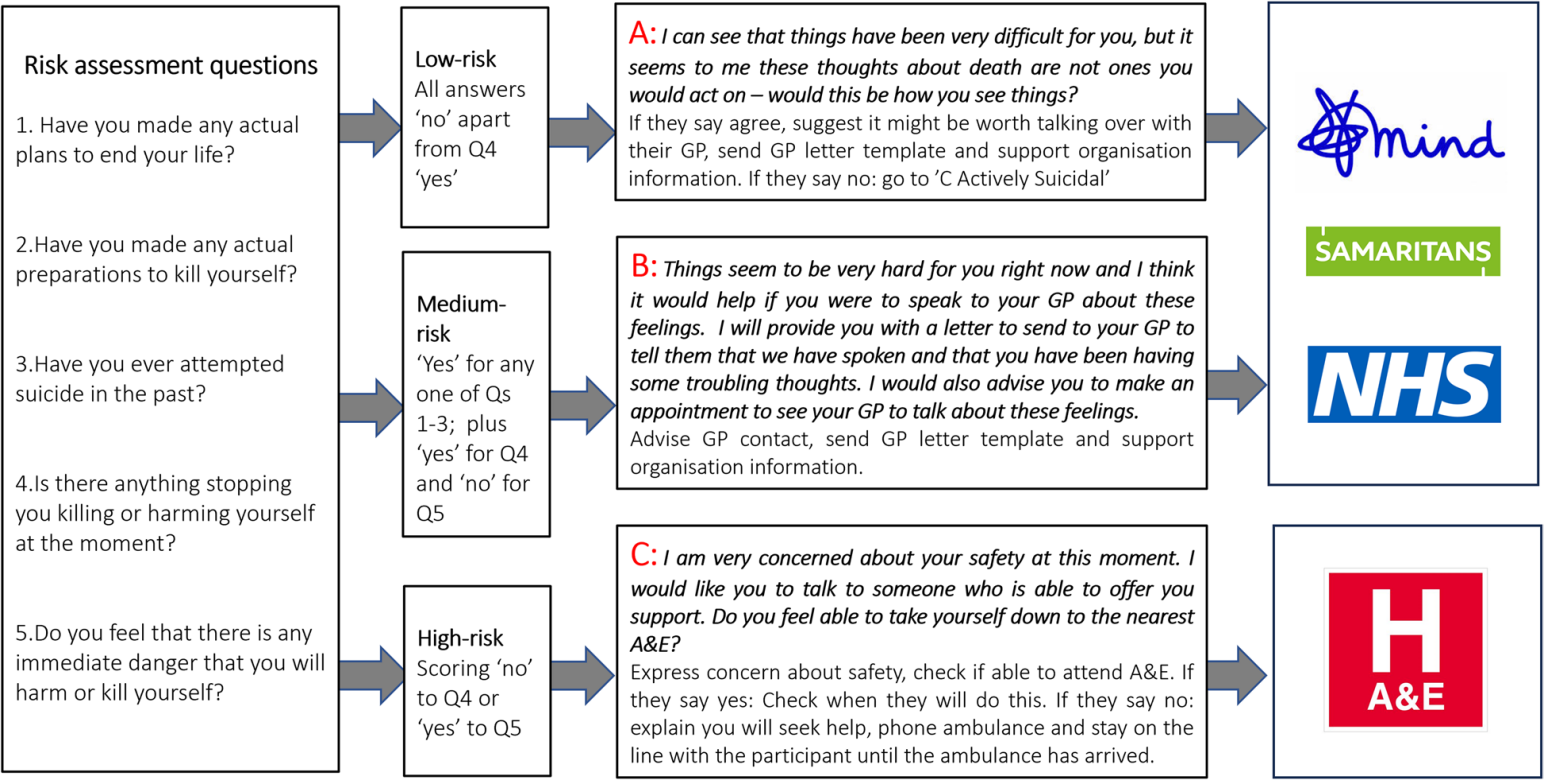
PHQ-9 risk assessment protocol evaluated in the trial

Participants completed the PHQ-9 scale at baseline and 6-month and 12-month follow-ups. Responding to the item 9: ‘*Thoughts that you would be better off dead or of hurting yourself in some way*’, if a participant indicated ‘*More than half the days*’ or ‘*Nearly every day*’ (see Fig. 1), the trial database automatically alerted the research team to risk assess the participant. The protocol specified that the participant be contacted by the research team within 10 working days of an alert and a PHQ-9 risk assessment initiated (see Fig. 2 and Additional file 1 for full protocol) if the participant consented.

All participants were assessed at the first occurrence of a PHQ-9 item 9 response alerted risk only. If a risk positive participant at baseline subsequently also alerted risk at 6-month and/or 12-month follow-up, the risk assessment was not repeated. If the alert response at 6 month or 12 months, was new, then a risk assessment was conducted. This decision was taken because the PHQ-9 risk assessment protocol was designed as a triage process at the point of the initial disclosed self-harm ideation and to signpost to appropriate resources and professional services as outlined above. It was also felt it might be intrusive, in the context of research trial participation, to contact the trial participant multiple times.

When a new risk was alerted, a member (research psychologist) of the research team, trained in the risk assessment protocol, called the participant using the telephone number they had provided. After ensuring the participant agreed to the assessment and was in a quiet, confidential space to carry out the assessment, the team member asked five questions (see Fig. 2). Depending on the participant’s responses, the researcher classified their level of risk as low, medium, or high. Participants assessed as low or medium risk were signposted to the UK mental health charity, Mind (mind.org.uk), and the UK suicide helpline charity, Samaritans (samaritans.org.uk) helpline with phone, text, and website information links, and advised to make an appointment with their NHS general practitioner (GP: family doctor) for a mental health review and to discuss their current feelings. They were also provided with a template letter to give to their GP if they wished, informing the GP of their participation in the trial and their self-reported self-harm ideation (see Additional file 1). For medium risk assessments, there was greater emphasis on encouraging the participant to contact their GP and seek help. If participants were assessed as high risk, they were asked if they were able to take themselves to their nearest accident and emergency (A&E) department. If they were unable to do this, an ambulance was to be called to their location whilst the researcher remained on the phone until it arrived. Signposting to charities and NHS GP would also be completed and follow-up contact with the participant would be made within 3 days.

If there was no answer to the initial telephone call, a brief, confidential message was left confirming we were calling from the IBD-BOOST trial team and would try again within 24 h. No reference to the questionnaire items or reason for the call was mentioned in these messages. If two calls remained unanswered, an email would be sent. If there was no response to the email, a letter would be sent (Additional file 1). All correspondence related to the protocol was documented in anonymous, password-protected documents, stored within secure trial databases. The frequency and outcome of PHQ-9 risk assessments was reported at monthly trial research team meetings and trial steering group meetings. PHQ-9 risk assessments at 6-month and 12-month follow-ups were reported as trial adverse events and reviewed by the chief investigator. If any adverse event was potentially related to the intervention, it would have been reported to the trial governance committees and sponsor, but this did not occur.

### Risk assessment training and support

An important part of the PHQ-9 risk assessment protocol for the IBD-BOOST trial was to consider the skills, training, and on-going support of the assessors. Members of the research team, with British Psychological Society accredited postgraduate degrees in Psychology, were trained to conduct the risk assessments. LS, PhD Research Psychologist, who utilised a similar protocol in a pilot study [28] was trained by RMM and then trained VW, PhD Research Psychologist, and SM, MSc Research Psychologist. Training included an initial meeting between the trainer and risk assessor to discuss the prevalence of depression in IBD and self-harm declaration with PHQ-9 item 9 responses and to review the risk assessment protocol. The risk assessor was required to familiarise themselves with the full protocol and to complete one practice call with the trainer, with feedback. Trial chief investigators, RMM and CN, provided supervision support throughout the trial to ensure the assessors could confidentially discuss risk assessments and debrief after stressful conversations, if needed.

### Evaluation of the PHQ-9 risk assessment protocol

To evaluate the PHQ-9 risk assessment protocol utilised in the IBD-BOOST trial, we collated quantitative data to report the frequency and outcome of assessments and supported this with qualitative data from the trial research team on their experiences of using the protocol. Summary data (mean and standard deviation [SD]), median, and range were measured for the PHQ-9 scale responses at baseline and 6-month and 12-month follow-ups, comparing all participants in the trial sample with those who were risk assessed. As one participant did not enter the trial for unrelated reasons, numbers of participants vary between summary PHQ-9 data tables and risk assessment tables. For PHQ-9 item 9, data were calculated to show the frequency (percentage) for each response item: ‘*Not at all*’, ‘*Several days*’, ‘*More than half the days*’, or ‘*Nearly every day*’. The number of risk assessments initiated, declined, and completed were recorded, including the number of repeated risk alerts. Risk assessment outcomes were analysed including time of call, risk category assigned, and action from the assessor. To explore the PHQ-9 risk assessment process from a research trial perspective, the risk assessment team (LS, VW, and SM) participated in a focus group, to reflect and discuss experiences of using the PHQ-9 risk assessment protocol in the IBD-BOOST trial. Using a semi-structured topic guide (Additional file 2), LM (trial manager) facilitated the focus group, which was audio recorded, transcribed, and analysed by LM.

## Results

The trial randomised 780 participants (mean age 48.5 years (SD = 14.4), 67% women (*n* = 524), from the 784 people who completed baseline assessments. Table 1 reports the frequency of PHQ-9 item 9 responses for all trial participants, illustrating the proportion of positive responses to ‘*Several Days*’ as well as those defined for the risk assessment criteria of ‘*More than half the days*’ or ‘*Nearly every day*’. PHQ-9 scale totals comparing participants by ‘at risk’ group are reported in Table 2. PHQ-9 total scores improved over time throughout the trial duration for those participants in the ‘not at risk’ group (MD = 1.2, *p* < 0.01). PHQ-9 total scores remained high for those participants who were still considered ‘at risk’ at 6-month and 12-month follow-ups.

**Table 1:** PHQ-9 item 9 responses

**PHQ9 item 9 responses**	*n* (%)	*n* (%)	*n* (%)	*n* (%)
	Not at all	Several days	More than half the days	Nearly every day
Baseline^a^	688 (88.2)	64 (8.2)	14 (1.8)	14 (1.8)
Six-month follow-up (*n* = 656)	586 (89.3)	52 (7.9)	12 (1.9)	6 (0.9)
Twelve-month follow-up (*n* = 466)	415 (89.1)	40 (8.6)	6 (1.2)	5 (1.1)

^a^Data reported for trial participants only (one trial participant assessed at baseline did not progress to trial)

**Table 2:** PHQ-9 total scores comparing ‘at risk’ and ‘not at risk’ participants

	**At risk**	**Not at risk**
**Baseline (*n* = 780)**	*n* = 28^a^	*n* = 752
Mean (sd)	21.3 (4.5)	8.8 (5.2)
**Six-month follow-up (*n* = 656)**	*n* = 18	*n* = 658
Mean (sd)	20.6 (4.0)	7.9 (5.2)
**Twelve-month follow-up (*n* = 466)**	*n* = 11	*n* = 476
Mean (sd)	21.5 (5.0)	7.6 (5.2)

^a^Data reported for trial participants only (one trial participant assessed at baseline did not progress to trial)

### PHQ-9 risk alerts and assessments

Overall, there were 58 PHQ-9 item 9 risk alerts during the trial (baseline and 6-month or 12-month follow-up), 41 of which were new alerts and required risk assessment (Table 3). The largest number of risk alerts was observed at baseline prior to randomisation (*n* = 29, 3.7% of 784 baseline questionnaires). All baseline alerts were assessed as low-risk or medium-risk, and all participants, except one who did not progress for unrelated reasons, progressed to trial randomisation.

**Table 3:** Number of PHQ-9 risk alerts and assessments throughout the trial

	**Baseline** (*n* = 784)	**6-month follow-up** (*n* = 656)	**12-month follow-up** (*n* = 466)	**Total**
PHQ9 risk alerts (*n*/%)	29 (3.7)	18 (2.7)	11 (2.3)	58
PHQ9 repeat risk alerts (*n*/%)	n/a	10 (1.5)	7 (1.5)	17
PHQ9 new risk alerts (*n*/%)	29 (3.7)	8 (1.2)	4 (0.9)	41
Risk assessments completed (*n*/%)	29 (3.7)	7 (1.0)^a^	4 (0.9)	40

^a^ One participant declined

Of 656 participants who completed 6-month follow-up PHQ-9 questionnaire, there were 18 (2.7%) risk alerts. Of these, eight were new risk alerts and seven assessments were completed, as one person declined. Due to COVID-19 delays, it was necessary to end 12-month follow-up data collection early, and therefore not all participants were invited to complete their 12-month questionnaires. Of the 636 participants who were invited at 12 months, 466 (73%) completed PHQ-9 outcomes measures, and of these, there were 11 (2.3%) risk alerts. Of these, seven participants had previously been assessed, and therefore, four risk assessments were completed. Five of the seven previously assessed participants alerted risk on all three occasions during the study (baseline and 6-month and 12-month follow-ups). Two participants had initially alerted risk at baseline, not at 6 months, but then were again at risk at 12 months. Overall, 40 risk assessments were completed; 24 participants were assessed as low-risk and 16 participants as medium-risk, with 12 declaring previous suicide attempts. No participants were rated as high risk at any time point.

The mean number of days for the study team to respond to risk assessment alerts was three days (range 0–15 days) highlighting that in one case the protocol response timeframe (within 10 days) was not followed as the first call attempt was made at 15 days. It was reported that this occurred due to research team absence. In most cases, there were no delays in reaching the participant to complete the risk assessment, except for two (4.5%) cases (48- and 52-day delay) where repeated attempts, as per the protocol, were made to contact, and in both cases, the participant eventually responded and was risk assessed. Only one participant declined risk assessment and did not provide a reason. Two participants declined receiving further information (GP letter/Mind and Samaritans details) in an email. The risk assessment calls were timed; data are missing for three calls. The total time spent on risk assessments calls (37 out of 40 timed calls) was 8 h and 55 min. The mean time for the risk assessment phone call was 14 min (range 5–36 min).

### Experiences of IBD-BOOST trial PHQ-9 risk assessment team

#### Training, expectations, and call preparation

In the focus group, members of the research team who assessed participants reflected on training experiences and expectations of making PHQ-9 risk assessment calls. They described finding it useful to familiarise themselves with the protocol and practise reading the script in a role-play call exercise. However, the assessment team recalled that they did not practice a Level C: high risk scenario where they would need to keep the participant on the phone whilst simultaneously calling for emergency services. Although no participants in our study were assessed as high risk, assessors explained that they were aware each time that this could have happened and would recommend that this is incorporated in future training:

But I think, maybe having like a role play session of what you actually have to do if that happens because I mean I know that it says “Oh, you call an ambulance and then you call a supervisor”. But realistically, how do you do that when you’re on also then on the phone to this individual especially when you’re like maybe working from home and you can’t.

Assessors recalled feeling nervous before conducting their initial risk assessments, but the training, script, and protocol helped guide them through the call and made them feel supported. They expressed various ways in which they prepared themselves for calls, from making sure their environment was suitable for the call (particularly privacy and confidentiality whilst working from home during the COVID-19 pandemic), wearing a headset to make sure hands were free to take notes, and writing preparatory notes/re-familiarising themselves with the protocol, prior to each call.

I think I just felt really nervous to make sure that everyone was out the house, the dog was quiet, you know, the doorbell wasn’t going to ring, you know, like, calm a sense of being. It would have been over prepared but that’s the trying to control all the knowns, when you knew you couldn’t control the unknowns.

### Experiences of conducting PHQ-9 risk assessment calls

Trial participants were called with no pre-warning and the assessment team discussed whether sending an email out beforehand advising of the call would have been useful, especially for those who may have been at work.

I found [it] sometimes quite tricky to do… you would ring them up….and they might be in the middle of like a working day. And I’m recalling some people they were at the office or one person I rang was a primary school teacher, you know, and then doing a really sensitive check in a phone call.

However, they recognised that this would have taken time, and the procedure and script was constructed to gradually introduce the reason for the call, to allow the participant to confirm whether it was convenient time for them and whether they were in a private space before sensitive questions were asked. They also recognised that the protocol and script helped to safely close the call, leaving the participant with options to follow-up resources with their GP or charities.

The way it [the script] was drafted in terms of just checking on people’s safety felt like a really nice thing to do…we’re just going through the responses and we’re wanting to check all of our participants are safe and well supported. It is quite a nice introduction. I think that beginning bit is really essential to say do you have time to speak, are you happy for us to proceed with the call...like there’s just some really simple steps you can take in the first minutes of the call….

So, it’s sort of making sure that we don’t just hang up and they feel like we’ve just opened a sort of wound and not helped manage it I suppose but I feel umm… the script does also adequately address that to sort of open and close the call safely.

The team felt that most participants were grateful for the telephone call and that they appreciated someone checking in with them. Participants were willing and open to answering the five questions during the risk assessment, although one question (Q4) about protective factors (see Fig. 2) confused some participants, and assessors described explaining the question with prompts to aid understanding. Re-phrasing this question for future trials could be helpful. Some participants were not aware of the support resources (e.g. charities Mind and Samaritans) and valued receiving information about these.

There wasn’t much hesitancy, never uncomfortable silences. People were very willing to talk about their experiences, particularly when they were very difficult ones. That surprised me. I didn’t expect it to be as easy for people to say about their previous [suicide] attempts or concerns or plans or people have been quite strikingly open about their previous attempts.

But I did have quite a few where patients were like, thank you so much for calling. Like, I’ve never experienced this, taken part in a lot of studies or it just means a lot to know that people are kind of, you know, wanting to check in on you and especially those that maybe were having more recent kind of like new kind of thoughts around like suicide and self-harm and giving them those kind of resources.

The team explained that although the script and protocol worked well, the algorithm which led to the risk assessment outcome was sometimes difficult to work through whilst simultaneously listening and responding to the participant and recommended that this be revised for ease of use during calls.

But you know that kind of sense of I’ve got to kind of think while being really sympathetic and listening and saying the right thing while I’m trying to just quickly work out…medium risk or low risk.

When asked how they felt after conducting the risk assessment calls, the team agreed that most of the time they felt fine but sometimes needed to call their risk-assessment team colleagues and supervisors (RMM and CN) to help them offload if they had had a particularly upsetting or difficult call. Having the supervision model in place for reflections is considered vital in this process.

I feel like maybe not drained but like definitely kind of touched and a bit like or after the calls occasionally not all the time … sometimes I did feel a little bit upset maybe. And like a senior, a senior colleague on hand on the phone.

Overall, the experience of conducting PHQ-9 risk assessments was considered a positive one for both assessors and participants. However, the presentation of the protocol could be more user friendly to complete during the risk assessment call where the attention is listening to the participant whilst checking the responses and outcome of the assessment. Further training/practice in doing this dual role would be preferable in future studies.

## Discussion

This paper reports the development and evaluation of a risk-assessment protocol for responding to research trial participants who declare thoughts of self-harm in a routine trial questionnaire (PHQ-9). The protocol was found to be feasible and not unduly onerous for the trial research psychologists to administer. Trial participants who received risk assessment appeared to appreciate being contacted and to find the protocol acceptable. We understand that many research trials might have their own protocols for risk assessment, but we have found none published; we share our protocol and evaluation to inform future research trials which utilise the PHQ-9.

In the IBD-BOOST research trial of 780 participants, 5.3% (*n* = 41) reported thoughts of self-harm at least ‘*More than half the days*’ in the previous 2 weeks, at some point during the trial. This is lower than a recent meta-analysis of the association between IBD and suicidal ideation, suicide attempts, and suicide, which reported a pooled prevalence of 17.3% suicide ideation in patients with IBD [29]. However, the measures varied and only two of the five included studies used the PHQ-9 item 9 to measure suicide ideation and counted participants who scored > 1 ‘*Several days*’, a lower threshold than ours. Had we included trial participants who recorded responses > 1, this would have meant 214 risk alerts (27.4%) throughout the trial and the feasibility study, and advice from IAPT colleagues supported a higher threshold for ‘at risk’ criteria. The variation might also reflect differences with our trial participants compared with general IBD population, i.e. those interested and willing to engage in a self-directed, psychological theory-based intervention programme like IBD-BOOST.

Of the 40 participants who were assessed, the majority (63%) were low risk and the remainder medium risk; there were no high-risk outcomes. Medium risk was determined by any positive response to risk assessment questions about previous suicide attempts and plans to end their life, whereas low-risk participants, although expressing thoughts of self-harm, did not report any previous attempts or plans. The language used by the risk assessor was different for low or medium risk. For low risk, the assessor summarised and confirmed the risk that the participant would act on the reported thoughts was low, whereas for medium risk, the risk assessor acknowledged the previous attempts and/or plans. However, the recommended actions were the same for both risk levels, and therefore, we reflected on whether the protocol should be revised to a two-tier risk level. However, we discussed that the distinction between low and medium was important to recognise and, instead, revised the protocol to further differentiate the recommended actions for each tier. For those assessed as medium risk, the risk assessor would express greater concern for the participant, and specifically ask questions to confirm if they will make an appointment with their GP, with an escalation response to high risk if necessary.

The trial research risk assessment team described initial concerns about calling participants to discuss their disclosure of self-harm or suicidal ideation with no prior notification and recalled being unsure how participants might respond. However, it is reassuring for future trials, which might wish to use the protocol, to learn that only one person declined risk assessment. Furthermore, the risk assessment not only appeared acceptable to trial participants, but many expressed how much they appreciated the contact, the care, and the information provided. In many instances, the risk assessment team described how many participants wanted to talk about their thoughts and feelings, and whilst risk assessors were careful to maintain their research role (not a therapeutic one), all recalled how they were happy to listen, show empathy, and give their time to the participant.

In the IBD-BOOST trial, three trial research team members with a research psychology background (MSc/PhD qualified) undertook the role of PHQ-9 risk assessors. This was a pragmatic decision in sharing trial tasks across the whole research team, but any healthcare staff in research trials could be trained to complete the risk assessment, as the PHQ-9 is routinely used in NHS healthcare settings and previous studies have demonstrated nurses’ competence in suicide risk assessment [30]. Resources within research trials are always limited and time should be costed for this purpose. Whilst this study found that the risk assessment phone call average time was relatively brief (14 min), time is still required to prepare for contact, complete trial documentation e.g. adverse event forms, and prepare follow-up communications. Furthermore, in some instances, risk assessors sought support from supervisors to reflect on their call experiences and this time needs to be accounted for as well as initial training time. It is also important for the trial to consider the need for supervision support and availability of supervisors. In the IBD-BOOST trial, there was no occurrence of supervision support not being available when required, but depending on the structure and organisation of trials, it might be appropriate to consider a more formal supervision process.

## Strengths and limitations

The IBD-BOOST trial is the largest trial of its kind, a cognitive behavioural self-management intervention to help improve multiple IBD symptoms (pain, fatigue, and faecal incontinence/urgency). It was important to recognise the likelihood of trial participants experiencing symptoms of depression, common in all LTCs, and to provide a process to triage participants to support. The inclusion of the PHQ-9 scale in pre-randomisation and follow-up questionnaires ensured that all participants, both intervention and control group, were included in the process. The PHQ-9 risk assessment protocol was developed prior to the updated NICE guidelines for *Self-harm: assessment, management and preventing recurrence* [19]. It is reassuring that the protocol is closely aligned with the guideline’s recommendation for *Assessment and care by professionals from other sectors* (those outside of mental health) particularly in treating the person with ‘respect, dignity and compassion’, including the person’s views about their situation and appropriate actions, referring to appropriate professional support services and incorporating an ‘immediate risk’ procedure.

One consideration was the decision to only risk assess each participant once, at the first instance they declared thoughts of self-harm, especially when five participants reported these thoughts on three occasions. The protocol decision was a pragmatic one, but it was also to maintain the boundaries of a research trial, and the outcome of the risk assessment protocol was to refer the participant to professional support, not to engage in a therapeutic role. We were also unsure how intrusive repeat calls might have been for trial participants in the context of a research trial they had volunteered for. However, future trials might wish to reflect on this decision and may consider repeated risk assessments. A further limitation is the follow-up process in the protocol, where we did not formalise the process and documentation of any follow-up. Because of this, we did not have follow-up outcome data, e.g. number of participants who contacted their GP and potentially related adverse events such as suicide attempts. In the future, we would recommend a more structured follow-up process, defining the method of contact, e.g. phone call or email, and recording outcomes. This is reflected in the updated version of the protocol (Additional file 1). Similarly, although the team recalled seeking support from supervisors for a small number of cases, the frequency and time spent on support calls from supervisors was not formally documented, and on reflection, this might have been helpful to measure. Finally, we did not ask participants who were risk assessed for their views on the process; we only had the assessor’s perceptions of acceptability.

## Conclusions

This study demonstrates that conducting a risk-assessment protocol for trial participants reporting thoughts of self-harm is feasible and acceptable. Participants who were risk assessed welcomed support and referral to further support services. Although we evaluated the risk-assessment protocol with participants who have IBD, the protocol is appropriate for use in any research programme where participants complete validated scales assessing depression. The research trial team was able to conduct risk-assessments with training and support from senior colleagues. Training should be comprehensive and cover all risk scenarios. Minor modifications were made to the evaluated protocol and the revised version is made available with this manuscript.

### Supplementary Information

Additional file 1. PHQ-9 risk assessment protocol.

Additional file 2. PHQ-9 risk assessment focus group topic guide.

## Data Availability

The data that support the findings of this study are available from the corresponding author on reasonable request.

## Abbreviations

CBA: Cognitive behavioural approach
CBT: Cognitive behaviour therapy
DSM-IV: Diagnostic and Statistical Manual of Mental Disorders
GP: NHS general practitioner
IAPT: Improving Access to Psychological Therapies
IBD: Inflammatory bowel disease
ISRCTN: International Standard Randomised Controlled Trial Number
LTC: Long-term conditions
NHS: National Health Service
NICE: National Institute of Health and Care Excellence
NIHR: National Institute for Health Research
PHQ-9: Patient Health Questionnaire

## Footnotes

1 It should be noted, however, that recently updated NICE guidelines do not recommend use of suicide risk assessment tools and scales to predict future suicide in decision-making about patient treatment for diagnosed depression, as it could lead to an inaccurate assessment [19].
