# Supplementary material for: Development and preliminary evaluation of a suicidal risk assessment protocol in a randomised controlled trial using the Patient Health Questionnaire (PHQ-9)
Source: Trials. 2024 Jul 12;25:476. doi: 10.1186/s13063-024-08276-6 (PMC11241891; doi:10.1186/s13063-024-08276-6)
Supplement: Supplementary file 1 — Additional file 1. PHQ-9 risk assessment protocol. [file 13063_2024_8276_MOESM1_ESM.docx]

**THE IBD-BOOST PROTOCOL FOR ASSESSING PHQ-9 SELF-HARM RISK RESPONSES IN RESEARCH TRIALS**

The Patient Health Questionnaire (PHQ-9) [1], a measure of depressive symptoms, includes a question (Item 9) which asks about suicidal thoughts and ideation. This protocol was developed to respond to trial participants sensitively and appropriately, to provide them with information and to signpost to professional help. The protocol was evaluated in The IBD BOOST trial [ISRCTN Registry ISRCTN71618461] and is reported in the accompanying publication (add citation).

PHQ-9 Item 9: *‘Thoughts that you would be better off dead or of hurting yourself in some way’*. There are four responses indicating the frequency of these thoughts in the past two weeks. If a participant responds with either of the two most frequent occurrences of these thoughts (*‘More than half the days or nearly every day’*), the participant is contacted by the [INSERT research team] within ten working days and a risk assessment undertaken. The risk assessment protocol is completed once, for a new report on this item during a study. The following principles and procedures govern risk assessment and reporting.

**A. General procedures**

Whenever any significant risk is identified, a risk assessment should be completed (and counter signed) by the Chief Investigator and/or nominated deputy) as soon after the assessment as possible.

Any significant, but not imminent risk should be reported to the person’s GP by the participant and, if appropriate, to other health care professionals, as soon as is reasonably possible.

Any imminent risk should lead to the immediate involvement of the appropriate emergency health services. The Chief Investigator must ensure that the [INSERT research team] are fully informed and competent to follow the procedures.

When the [INSERT research team] members who carried out this risk assessment are absent they should ensure appropriate cover is arranged for any risk issues that might arise in their absence.

**B. Protocol for contacting trial participants**

The [INSERT research team] member will telephone the participant. If no answer, a brief message can be left confirming that they are calling from [INSERT] trial team and will try again within 24 hours. No reference should be made to the questionnaire items or reason for their call. If two calls remain unanswered, then an email can be sent. If the email is not responded to within 7 days, a letter can be sent. Email/Letter template below.

**C. Phone call script**

*“Hello ****, my name is **** I’m calling from* [INSERT research team] *on behalf of the* [INSERT research study name] *study. Is now a good time to talk? (If not, ask when would be convenient to phone back). Before we begin, may I please check that you are somewhere quiet where you are able to speak freely?*

*Thank you for your involvement in the study so far and for completing the study questionnaire. As part of the research process, we review questionnaire responses. One of the questions asks about thoughts of self-harm and I see that you’ve responded positively to the question. I am just you as it’s important that we ensure that people taking part in the [INSERT research study name] have the right support and aren’t at any immediate risk to themselves. These are thoughts / feelings that people suffering from*

*depression could experience and can be very distressing, therefore it’s important to make sure you are receiving the right kind of support. I appreciate that this is a very sensitive area to be discussing, if you feel things are becoming too much then we can stop and take a breath then continue. I would now like to ask you some more questions that will explore these feelings in a little more depth. Would that be okay?*

If participant is happy to go through the questions, then please continue to **D. Risk assessment questions** below.

If participant declines to give any more information, then please offer some reassurance again. *“Is there a particular reason you don’t want to continue with the call/give more information at present?”* If it is due to environment, offer another time to call back. If they don’t wish to discuss it further, please check with them that they are aware of how to seek support.

“*I can appreciate that this is a difficult conversation to have with someone, I am keen for you to be given the opportunity to get the right support. Please could I give you details of some services which could help?” We can also send these via post or email*” *I would also advise you see your GP to make an appointment as they will have links to services which can support you during this time and put things in place to help you. I can provide you with a template letter to your GP to inform them of our discussions. If you find that the thoughts are become intense and you’re concerned about the risks to yourself, please take yourself to the nearest A&E departments where you would be seen by a psychiatric team who would be able to support you.”*

**D. Risk assessment questions**

**If the participant is happy to continue with the phone call. Ask the 5 questions below.**

**1. Have you made any actual plans to end your life? Yes/No**

**If yes?** Are you able to tell me more about this? What do you plan to do? When do you plan to do this? *Thank the person for offering the information*

**2. Have you made any actual preparations to kill yourself? Yes/No**

**If yes?** Are you able to tell me what preparations you’ve made? *Thank the person for offering the information*

**3.Have you ever attempted suicide in the past? Yes/No**

**If yes?** When was this? What did you do? *Thank the person for offering the information*

**4.Is there anything stopping you killing or harming yourself at the moment? Yes / No**

**If yes?** Are you able to tell me what stops you? Is there something you think about at the time?
*Thank the person for offering the information*

**5.Do you feel that there is any immediate danger that you will harm or kill yourself? Yes / No**

**If yes?** In what way? What do you think will happen? *Thank the person for offering the information and move onto the actions dependant on their response.

**E. Researcher response and action**

| **Participant response** | **Level of risk** | **Researcher response** |
| --- | --- | --- |
| Q1: No  Q2: No  Q3: No  Q4: Yes  Q5: No | **A**  **Low** | I can see that things have been very difficult for you, but it seems to me these thoughts about death are not ones you would act on – would this be how you see things? **If they say yes:** I would advise you to make an appointment to see your GP to talk about these feelings and I can provide you with a template letter to your GP to inform them of our discussions. **If they say no:** go to C (high) |
| Q1: Yes or No  Q2: Yes or No  Q3: Yes or No  Q4: Yes  Q5: No | **B Medium** | Things seem to be very hard for you right now, I am concerned about you, and I strongly recommend that you speak to your GP about these feelings. I will provide you with a letter to send to your GP to tell them that we have spoken and that you have been having some troubling thoughts. Do you feel able to make an appointment to see your GP to talk about these feelings? **If they say yes:** Check when they will do this. **If they say no:** reiterate your concern and ask if there’s any reason why they cannot make an appointment with their GP – use your judgement to signpost to Mind and Samaritans or to escalate response to C (high). |
| **For risk levels A (low) and B (medium) complete the [INSERT NON-SERIOUS ADVERSE EVENT TRIAL FORM]. Send information sources and GP template letter to participant. If appropriate, ask the participant if they would like a follow-up call within one week.** | | |
| Q1: Yes or No  Q2: Yes or No  Q3: Yes or No  Q4: No  Q5: Yes | **C**  **High** | I am very concerned about your safety at this moment. I would like you to talk to someone who is able to offer you support. Do you feel able to take yourself down to the nearest A&E? **If they say yes:** Check when they will do this, and signpost participant to Samaritans helpline (**116 123**). **If they say no*:*** I am going to contact someone to let them know how you are feeling and to arrange for you to receive immediate help. Could I check where you are so that I can arrange for an ambulance to come to you? Please stay on the line as I am still here and available. **Do not leave them alone**. Phone ambulance and stay on the line with the participant until the ambulance has arrived. Once you have confirmed that the ambulance has arrived and that the participant is receiving help complete research documentation. |
| **For risk level C (high) complete the write a report of the call and the outcome on the [INSERT APPROPRIATE SERIOUS ADVERSE EVENT (SAE) TRIAL FORM]*.* Notify the Chief Investigator [INSERT NAME] via phone on [INSERT PHONE NUMBER] if possible, or via email [INSERT NHS EMAIL ADDRESS] and forward a copy of the SAE via NHS email. Ensure to follow-up with the participant within 3 working days in cases of immediate risk.** | | |

**E: Information sources and templates**

Promotes the views and needs of people with mental health problems. Phone: **0300 123 3393** or Text: **86463** (9am-6pm, Mon-Fri, except for bank holidays). [**www.mind.org.uk**](http://www.mind.org.uk)

**
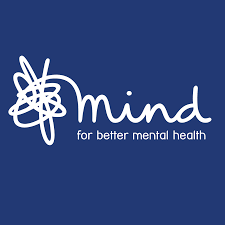
**

Confidential support for people experiencing feelings of distress or despair.

Phone: **116 123** (free 24-hour helpline)

**www.samaritans.org.uk**


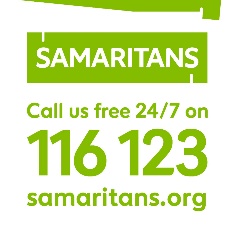


**Participant email/letter template (adapt depending on study timepoint (baseline/follow-up) and contact attempts)**

1. Thank you for your interest in the [INSERT RESEARCH STUDY]

at [INSERT STUDY SETTING] and for completing the baseline questionnaire. As part of the research process, we review responses from the questionnaire to check all participants are receiving the right kind of support for them. It would be helpful if we could ask you a few further questions, before entry into the [INSERT RESEARCH STUDY].

1. Thank you for your participation in our research trial [INSERT RESEARCH STUDY] for completing the [INSERT TIME POINT] questionnaire. I have tried contacting you by phone regarding your completed survey and as I couldn't leave a message, I thought it might be best to send you an email. Is there a convenient time for me to call you, or if you prefer, you can call me on [INSERT PHONE NUMBER] or the [INSERT RESEARCH STUDY] trial phone number.
2. Thank you for your interest/participation in the [INSERT RESEARCH STUDY]

at [INSERT STUDY SETTING] so far and for completing the [INSERT TIME POINT] questionnaire. We appreciate you taking the time to participate in [INSERT RESEARCH STUDY]. As part of the research process, we review responses from the questionnaire to check all participants are receiving the right kind of support for them. I have tried to call and email but as we haven’t been able to speak yet, I wanted to write to you to get in touch. I suspect you may be away so my apologies for these multiple attempts, but so we don’t miss you, I would be most grateful if you could either email me [INSERT EMAIL ADDRESS] to let me know if there’s a convenient time to speak or call [INSERT PHONE NUMBER]

**GP letter template (to be sent to participant)**

Date:

Private & Confidential

To be opened by addressee only

Dear Dr ________________,

I am currently taking part in the [INSERT RESEARCH STUDY], which is a research study looking at whether an online self-management programme can improve [INSERT ILLNESS SYMPTOM e.g., pain and quality of life in people with [INSERT CONDITION/DIAGNOSES].

As part of this study, I completed a questionnaire about my feelings and expressed having had thoughts of suicidal ideation. The research team contacted me on dd/nn/yyyy to discuss this with me further and assessed me as [INSERT RISK LEVEL low/medium/high risk]. I was therefore advised to see my GP and given contact details of Mind and Samaritans/advised to take myself to A&E/ and see my GP/but as I didn’t feel able to, the research team phoned for an ambulance [AMEND/DELETE AS APPROPRIATE].

The study research team advised that I see you for a review as soon as possible, and gave me the following information:

Mind Promotes the views and needs of people with mental health problems.

Phone: 0300 123 3393 or Text: 86463 (9am-6pm, Mon-Fri, except for bank holidays).

Website: www.mind.org.uk

Samaritans Confidential support for people experiencing feelings of distress or despair.

Phone: 116 123 (free 24-hour helpline)

Website: www.samaritans.org

If you would like to contact the research team, their details can be found on the study website: [INSERT RESEARCH STUDY WEBSITE OR ALTERNATIVE CONTACT DETAILS]

**References**

1. Kroenke, K., & Spitzer, R. L. (2002). The PHQ-9: a new depression diagnostic and severity measure. Psychiatric annals, 32(9), 509-515.
