# Supplementary material for: Development and preliminary evaluation of a suicidal risk assessment protocol in a randomised controlled trial using the Patient Health Questionnaire (PHQ-9)
Source: Trials. 2024 Jul 12;25:476. doi: 10.1186/s13063-024-08276-6 (PMC11241891; doi:10.1186/s13063-024-08276-6)
Supplement: Supplementary file 2 — Additional file 2. PHQ-9 risk assessment focus group topic guide. [file 13063_2024_8276_MOESM2_ESM.docx]

**Topic guide (PHQ-9 risk assessment study)**
**Training**

Which team members were mostly involved in carrying out the risk assessments?

Prompts How did you decide this?

How useful did you find the training session?

Prompts Did it cover everything you needed to know? Were you left with unanswered questions or concerns? What other information would you have liked?

**Experiences of using protocol**

What were your experiences of conducting the risk assessments with patients?

Prompts What were your expectations before carrying out a risk assessment?

Did you have any worries before carrying out a risk assessment? What preparations were made before each risk assessment call?

What kind of feedback/response did patients give you about their risk assessments if any?

Prompts How able were patients to answer the questions in the risk assessment?

Were there any patients that refused to answer questions?

Was there a time you were unable to contact a patient about their PHQ-9 risk assessment?

Prompts What did you do?

How easy was it to understand the content of the protocol?

Could any aspects of the protocol presentation be improved?

Are there any other issues you wish to mention or discuss about the PHQ-9 risk assessment protocol that we haven’t already covered?
